# Neurotrophic keratitis caused by lightning injury: a case report

**DOI:** 10.1186/s12886-024-03512-8

**Published:** 2024-06-10

**Authors:** Sidou Yi, Guoping Wang, Xuan Meng, Xuejing Lu

**Affiliations:** 1 Chengdu University of Traditional Chinese Medicine, Sichuan Province, 37 Twelve Bridge Road, Chengdu City, China; 2 Ineye Hospital of Chengdu University of Traditional Chinese Medicine, Sichuan Province, Chengdu City, China

**Keywords:** Lightning injury, Neurotrophic keratitis, Cornea, Eye injury

## Abstract

**Background:**

This study aimed to report a case of neurotrophic keratitis caused by lightning.

**Case presentation:**

A 38-year-old man was hit by lightning and suffered eye injury. He eventually developed neurotrophic keratitis.

**Results:**

The patient’s injury history and burn site were analyzed, and it was judged that lightning directly damaged his cornea, eventually resulting in neurotrophic keratitis. Fortunately, the patient’s vision improved after treatment.

**Conclusion:**

Lightning can cause eye damage, and the clinical manifestations are diverse. Lightning currents cause corneal nerve loss, resulting in neurotrophic keratitis. To maintain corneal integrity and prevent disease progression, early assessment and appropriate treatment are necessary.

## Background

Lightning is a natural phenomenon with about 50 events per second on Earth. It is estimated that 240,000 people die from lightning strikes every year worldwide [1]. Lightning can cause injury or even death through direct strikes, contact injury, side flash, and step voltages [2]. Furthermore, it can cause multiorgan damage. Lightning injuries are rarely reported in the ophthalmic field, with the exception of cases of keratitis, cataracts, anterior uveitis, retinal detachment, and macular holes [3–5]. Additionally, optic nerve injury, papillitis, abnormal pupillary reflexes, and Horner syndrome have been documented in a limited number of cases [6, 7].

Lightning causes damage through a variety of forms. The factors of lightning injury include thermal, electrical, magnetic field, and explosive effects as well as blunt trauma due to falls. There is a way in which lightning strike can cause injury of the phenomenon known as flash-over. With flash-over, current travels over the skin and burns the body. Its burn wounds can include entrances and exits [8]. When the current enters the tissues of different resistance in various parts of the human body, a thermal effect occurs, transforming electrical energy into heat energy. Different types of tissues in the body have different resistance levels (from most to least resistance: bone, fat, tendon,skin, muscle, blood, nerves) [9]. Therefore, nervous tissue is very susceptible to thermal injury. Shockwaves can also cause injury to humans. The pressure reduces, eventually resulting in shockwave. This shockwave can cause rupture and damage to human tissues and organs as well as indirect damage to the human body.

The present study reports a rare case of neurotrophic keratitis resulting from a lightning injury. Neurotrophic keratitis is a blinding eye disease that arises when the corneal inflammatory response is activated by damage to the trigeminal nerve, changes in corneal nerve morphology, and a decrease in corneal neurosecretory function and sensibility due to a variety of causes. This results in a series of pathological changes, including corneal epithelial defects, delayed healing, and corneal ulceration. A review of the literature has revealed that all conditions that can cause damage to the Vth cranial nerve, from the trigeminal nucleus to the corneal nerve endings, can contribute to neurotrophic keratitis [10]. A multitude of studies have demonstrated that reduced tear secretion, diminished corneal neuroreceptivity, aberrant metabolism of epithelial cells, and a diminution or even loss of transmitters and neurotrophic factors secreted by corneal nerve fibres may all be potential mechanisms for the emergence of neurotrophic keratitis [11–14].

A diagnosis of neurotrophic keratitis is necessary for decreased corneal perception, in addition to a history of trigeminal nerve damage and the characteristic separation of signs and symptoms of the lesion. Some examination equipment, such as corneal confocal microscopy, can effectively assist clinicians in analysing the corneal nerve morphology and in the diagnosis. Corneal confocal microscopy represents the only currently available method for in vivo observation of corneal nerves, enabling the observation of all corneal structures, including the epithelium, basal plexus, keratocytes and endothelial cells. In patients with neurotrophic keratitis, changes in the number and density of corneal nerve fibres can be observed in vivo using corneal confocal microscopy to assess corneal nerve damage. This can then be used to inform clinical diagnosis and treatment.

In the early stages of neurotrophic keratitis, it is often not treated in a timely manner due to misdiagnosis and lack of attention by the patient, which ultimately leads to spontaneous corneal epithelial rupture, corneal ulceration or even perforation, with a poor prognosis. In terms of clinical practice, the majority of existing treatments are only capable of slowing the progression of the disease. There is, as yet, no effective cure. A comprehensive investigation of the disease's pathogenesis is of paramount importance for the development of effective treatment strategies and the enhancement of clinical therapeutic outcomes.

Neurotrophic keratitis resulting from a lightning strike is an exceedingly rare occurrence. The objective of this study was to investigate the potential mechanisms underlying neurotrophic keratitis following a lightning strike. To this end, we collected and analysed data from corneal confocal microscopy, with a particular focus on the impact of lightning strikes on corneal nerves.

## Case Presentation

**Fig. 1:**
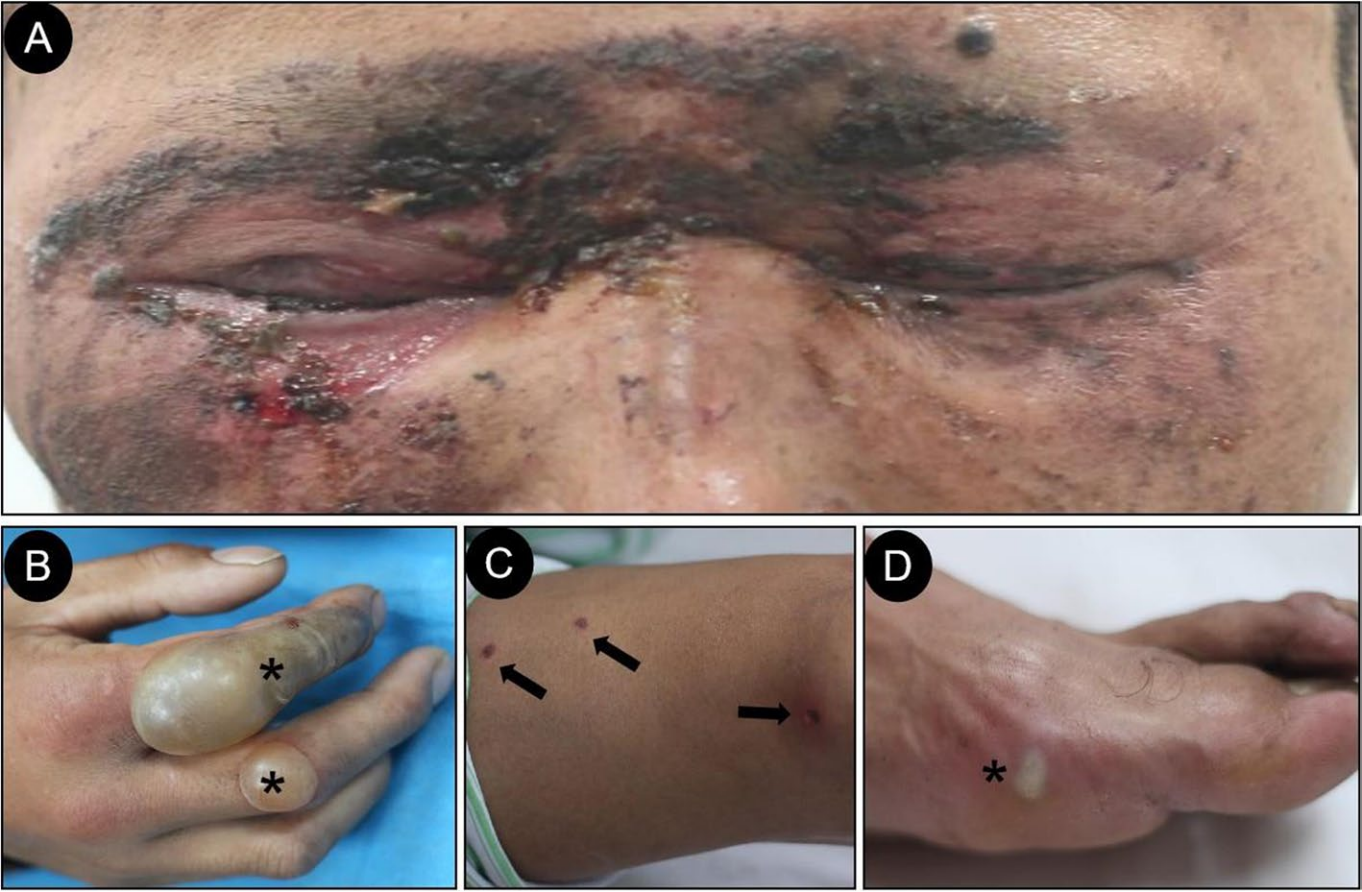
**A**: the entrance of lightning strike was found at the lower eyelid of right eye. **B**: the main exit was found at the extensor side of the index finger of the right hand. **C** and **D**: the rest of the exits were located at the elbow, knee joints and toes

**Fig. 2:**
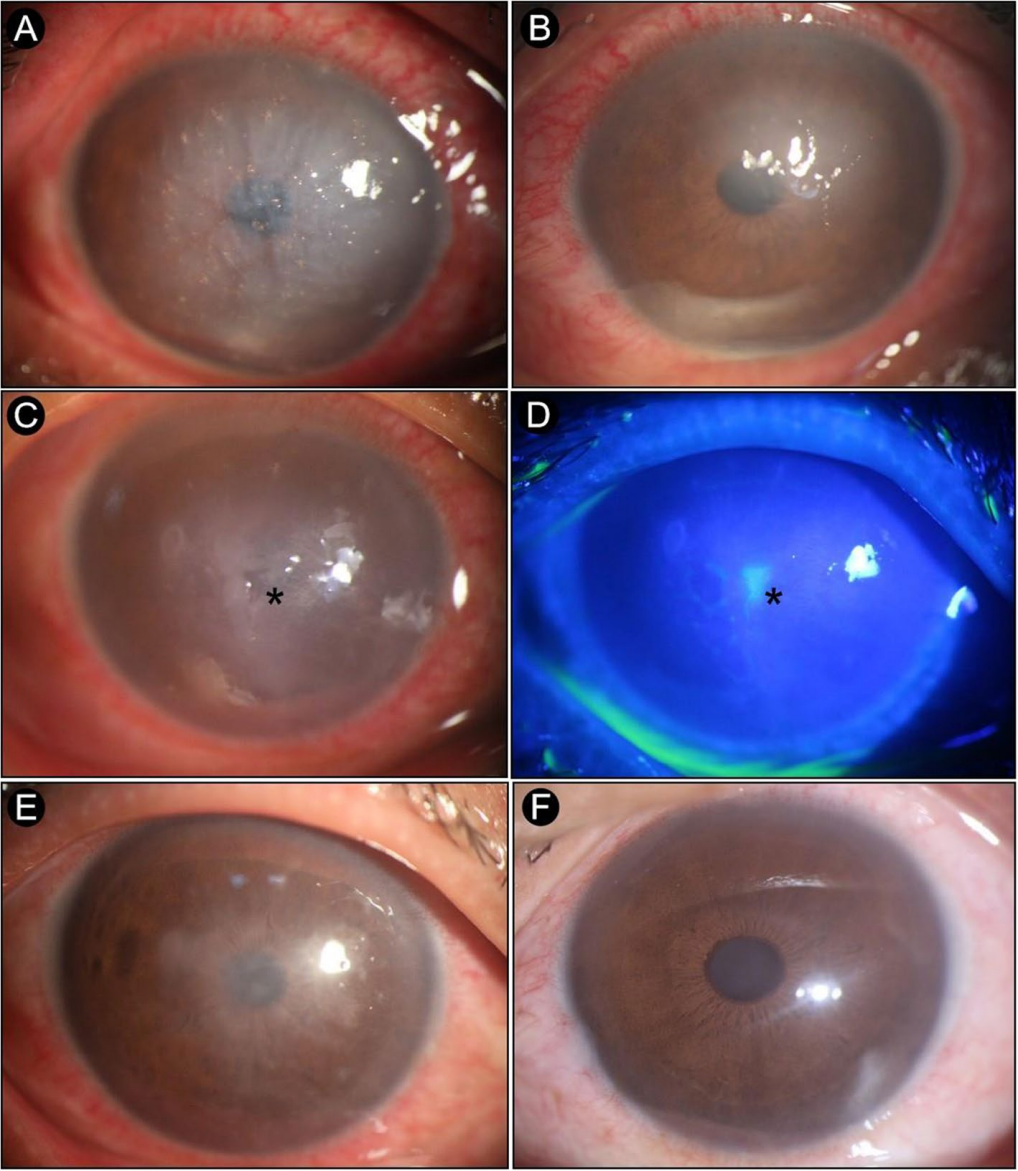
**A** (right eye): the central stroma of the cornea was edematous, and a large number of dotlike golden foreign bodies was observed in the shallow stroma. **B** (left eye): the central stroma of the cornea was cloudy. **C**: corneal sensation disappeared, and a small ulcerated lesion was found at the center of the right eye’s cornea measuring about 1 * 2.5 mm. **D**: fluorescent of the right eye’s cornea staining was positive. **E** (right eye): corneal nebula left in the center of the cornea. **F** (left eye): the cornea was largely transparent

A 38-year-old man was treated at our hospital for “pain in both eyes with vision loss for 2 days after being hit by lightning.” The patient was in good health prior to the injury and did not have any other systemic diseases. The patient lives in a highland pastoral area in China. He was struck by lightning in a tent. When he regained consciousness, he felt pain in both eyes, photophobia, and tearing, and his vision significantly reduced. In hospital, we found that his vital signs were stable, but he had multiple burns all over the body. Whole body examination revealed burns on the lower eyelid of his right eye as well as a large number of bands and flaky brown-black crusts and dark ecchymoses on his face, which was the entrance of the lightning strike (Fig. 1-A). The exits were at the distal end of the limbs, mainly in the index and middle fingers of the right hand. A blister with a height of about 3 cm and a size of about 6 * 3 cm was found at the extensor side of the index finger of the right hand from the distal segment to the proximal segment (Fig. 1-B). The rest of the exits were located at the elbow and knee joints, toes, etc. (Fig. 1-C, D). The patient’s index and middle fingers on the right hand were tender and swollen and also had limited movement. Neurological examination revealed no significant differences in the distribution area of the maxillary and mandibular branches of the trigeminal nerve and in the bilateral comparison of the upper eyelid and nasal skin sensation innervated by eye branches. Furthermore, no difference was observed in the muscle strength of the masticatory muscle group, and there was no skew in the lower jaw. The corneal reflexes of the right and left eyes were absent and reduced, respectively. The muscle strength of both lower extremities was grade IV (the Lovett grading standard). Specialist ocular examination(Table 1): Binocular visual acuity was hand motion in front of the eye, right eye intraocular pressure of 16 mmHg, left eye intraocular pressure of 18 mmHg. The inverted triangle-shaped skin under the right eyelid was lost, and hyperemia and edema were observed on the conjunctiva. The central stroma of the cornea was edematous, the turbidity range was about 8*8 mm, and a large number of dotlike golden foreign bodies was observed in the shallow stroma (Fig. 2-A). The Descemet’s membrane wrinkle was occured. The depth of the anterior chamber was normal, anterior chamber flare (+), anterior chamber cells (−). The pupils were equal, round, and reactive to light. The posterior segment of the eye could not be seen. Furthermore, the central stroma of the left eye’s cornea was cloudy (Fig. 2-B). The other parts had no obvious abnormalities. Anti-infection treatments were given. To eliminate inflammation, systemic intravenous antibiotic drip, topical antibiotic eye drops, tobramycin dexamethasone eye drops, and eye ointment were administered. For corneal repair, calf blood-deproteinized gel was given. Active mydriasis prevented pupil inflammatory adhesions. The burn wound was disinfected, and dressings were regularly changed.

**Table 1:** The Specialist ocular examination of the patient. And the clinical timeline of twice hospitalization was showed in this table

The Specialist ocular examination and the clinical timeline of twice hospitalization								
	First hospitalization				Second hospitalization			
Specialist ocular examination	onset		10 days of treatment		onset(One week after discharge)		10 days of treatment	
	OD	OS	OD	OS	OD	OS	OD	OS
visual acuity	Hand motion	Hand motion	20/167	20/20	CF/5 cm	20/25	20/100	20/25
Intraocular pressure	16 mmHg	18 mmHg	normal	normal	normal	normal	normal	normal
corneal reflexes	absent	reduce	/	/	absent	reduce	/	/
cornea	edema, Descemet’s membrane wrinkle	edema	Edema was alleviated	small corneal nebula	small ulcerated lesion	small corneal nebula	Corneal nebula left	largely transparent
anterior chamber	anterior chamber flare (+)	anterior chamber flare (+)	anterior chamber flare (-)	anterior chamber flare (-)	normal	normal	normal	normal
pupils	normal	normal	normal	normal	normal	normal	normal	normal
posterior segment	can ‘t be seen	can ‘t be seen	normal	normal	normal	normal	normal	normal

After 10 days of treatment, the patient’s eye condition improved. VOD, 20/167; VOS, 20/20. Edema of the cornea in the right eye was alleviated, but the corneal epithelium was not visible. Some interlaminar foreign bodies were spontaneously discharged. A small corneal nebula remained above the left eye. There were no obvious abnormalities in the posterior segment of both eyes. Electric shock wounds in the face and body improved. Eventually, the patient was discharged from the hospital with a corneal bandage lens placed on his right eye for treatment continuation.

**Fig. 3:**
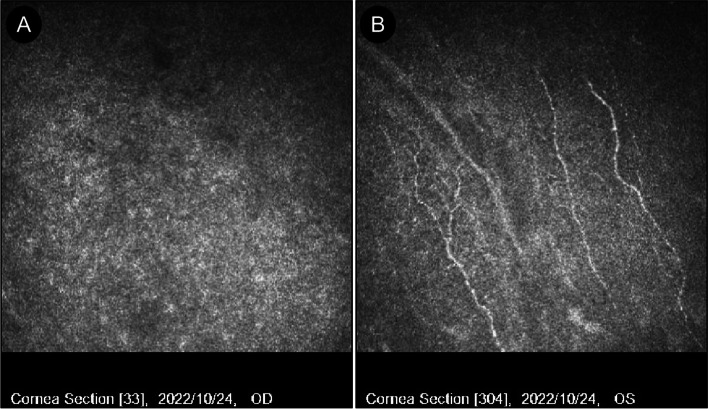
**A** (right eye): corneal confocal microscopy revealed that nerve fibers were absent. **B** (left eye): the density of subbasal nerve fibers was significantly reduced, and some nerves were bead-like

One week after discharge, the patient experienced burning pain, photophobia, tearing, and decreased vision in his right eye. He also lost the bandage lens after rubbing his eye. He was hospitalized again. Eye examination revealed VOD of CF/5 cm and normal intraocular pressure. Corneal sensation disappeared, and a small ulcerated lesion was found at the center of the right cornea measuring about 1 * 2.5 mm (Fig. 2-C); fluorescent staining was also positive (Fig. 2-D). Corneal confocal microscopy revealed that nerve fibers were absent in the right eye(Fig. 3-A) but present in the left eye. However, the density of subbasal nerve fibers was significantly reduced, and some nerves were bead-like(Fig. 3-B). There was no pathogen in either eye; thus, we ruled out fungal and amoebic infections. Neurotrophic keratitis was diagnosed based on past medical history, examination findings, and clinical signs. Levofloxacin eye drops were administered to prevent infection, cyclosporine eye drops to suppress immune and anti-inflammatory reactions, bovine alkaline fibroblast growth factor eye drops and calf blood-deproteinized extract ophthalmic gel to promote corneal repair, and tetracycline eye ointment with anti-collagenase to prevent corneal lysis. Movable pupil therapy was also given. At the same time, oral inosine, methylcobalamin, complex vitamin B, and vitamin C injection were administered to nourish the nerves. After 10 days of treatment, the visual acuity values were 20/100 and 20/25 in the right and left eyes, respectively. Corneal nebula left in the center of the cornea of the right eye (Fig. 2-E); The cornea in the left eye was largely transparent (Fig. 2-F).

## Discussion

Eye damage caused by lightning is rare. In recent decades, there were few reports of keratitis, cataracts, uveitis, and macular damage caused by lightning strikes. We herein report a case of neurotrophic keratitis caused by lightning strikes. Neurotrophic keratitis is an eye disease that causes blindness. It is caused by disruption of normal corneal innervation. Neurotrophic keratitis caused by lightning strikes is an extremely rare case. A review of previous literature revealed that lightning can cause corneal oedema and corneal damage [15]. However, the extent of corneal damage and the most appropriate treatment were not documented in sufficient detail [15]. This article analyzes the possible mechanism of neurotrophic keratitis from the perspective of corneal nerve injury due to lightning strikes. In this case, the changes in corneal nerve fibres following a lightning strike were observed dynamically using corneal confocal microscopy, with the aim of providing a reference for the diagnosis of neurotrophic keratitis due to specific causes.

In the present case, the eye damage was mainly caused by the thermal effect of lightning currents as well as shockwaves. The patient’s injury site was examined, and it was judged that the lightning current entered through the lower eyelid of the right eye and the surrounding skin muscles were burned by the thermal effect and currents conducted along the skin to the ends of the limbs in the form of flashes. It was also deduced that the foreign body was from a burning debris in the tent with the lightning shockwave embedded in the cornea. The position of the foreign body in the corneal layers was relatively shallow, and it gradually discharged by itself during hospitalization; thus, no special treatment was given.

All conditions that can cause damage to the Vth cranial nerve, from the trigeminal nucleus to the corneal nerve endings, can contribute to the development of neurotrophic keratitis. Corneal sensory nerve fibers are derived from the ophthalmic branch of the trigeminal nerve (cranial nerve V). Corneal nerves release neurotransmitters and nutritional factors that provide nutritional support to ocular surface tissues and promote wound healing. Damage to corneal nerve fibers can lead to corneal diseases [16]. The pathological changes of corneal nerve damage mainly include the following three aspects: corneal neuromorphological abnormalities (decreased nerve fiber density, reduced branches, shortened length, tortuous nerve fibers, neuroma formation, and bead-like changes) [17], decline in corneal neurosecretion function (neurotransmitters and nutritional factors released by corneal nerve fibers activate each other, and any disease causing corneal sensory nerve damage can impair its function, causing further damage to the cornea) [18], and reduced corneal neuro-susceptibility (decreased blink reflex and impaired tear film stability, which negatively affects the removal of harmful substances from the eyes and the inflammatory mediators in the tear film as well as aggravates the degree of damage to the corneal epithelium).

In the present case, neurological examination revealed no obvious abnormalities in the trigeminal nerve innervation area, except for the cornea, and it was judged that the trigeminal nerve was not directly damaged by the lightning current. Furthermore, corneal confocal microscopy revealed loss of corneal nerves in the right eye and reduced density of the subbasal nerve fibers in the left cornea; some nerves were also beaded. It can be inferred that in this patient, the corneal nerve damage was caused by direct damage to corneal nerve fibers by lightning. In this case, the lightning produced a thermal effect. The tissues around the entrance were severely burned, and the corneal nerve fibers were damaged or even disappeared. Abnormal corneal nerve morphology results in reduced corneal neurosecretory function and a reduction or loss of the transmitters and neurotrophic factors, eventually leading to neurotrophic keratitis.

In this case, the diagnosis of neurotrophic keratitis was confirmed by analysing the mechanism of lightning injury. Preservative-free artificial tears and drugs promoting corneal repair and nutritional effects were administered to maintain the surface of the cornea in a moist state and facilitate epithelial repair. Additionally, an antibiotic ointment was applied to the conjunctival sac prior to bedtime to prevent secondary infections. Concurrently, a therapeutic corneal bandage lens was utilized to safeguard the cornea and maintain moisture, which mitigated mechanical friction on the eyelids and facilitated the repair of corneal lesions. After the administration of symptomatic treatment, the patient’s prognosis improved, and the disease did not recur.

## Conclusions

We herein present a case of neurotrophic keratitis with a rare etiology and discuss how lightning injury leads to neurotrophic keratitis. Lightning is a rare cause of accidental injury, which results in eye tissue damage, and its clinical manifestations are diverse. When treating lightning injuries, in addition to the general condition, possible eye damage should also be considered. In the present case, lightning currents caused corneal nerve loss, which led to neurotrophic keratitis. To maintain corneal integrity, prevent disease progression, and save the patient’s vision to the greatest extent, early assessment and appropriate treatment are necessary. At the same time, follow-up should be maintained for possible cataracts several years later.

## Data Availability

No datasets were generated or analysed during the current study.

## References

[1] Davis C, Engeln A, Johnson EL (2014). Wilderness medical society. wilderness medical society practice guide-lines for the prevention and treatment of lightning injuries: 2014 update. Wilderness Environ Med.

[2] Edlich RF, Farinholt HM, Winters KL (2005). Modern concepts of treatment and prevention of lightning injuries. J Long Term Eff Med Implants.

[3] Venkateswaran N, Galor A (2018). Rosette-shaped cataract due to lightning injury. JAMA Ophthalmol.

[4] Rao KA, Rao LG, Kamath AN (2009). Bilateral macular hole secondary to remote lightning strike. Indian J Ophthalmol.

[5] Sommer LK, Lund-Andersen H (2004). Skin burn, bilateral iridocyclitis and amnesia following a lightning injury. Acta Ophthalmol Scand.

[6] Moran KT, Thupari JN, Munster AM (1986). Lightning injury: physics, pathophysiology and clinical features. Ir Med J.

[7] Lee MS, Gunton KB, Fischer DH (2002). Ocular manifestations of remote lightning strike. Retina.

[8] Muehlberger T, Vogt PM, Munster AM (2001). The long-term consequences of lightning injuries. Burns.

[9] Christensen JA, Sherman RT, Balis GA (1980). Delayed neurologic injury secondary to high-voltage current, with recovery. J Trauma.

[10] Hsu HY, Modi D (2015). Etiologies, quantitative hypoesthesia, and clinical outcomes of neurotrophic keratopathy. Eye Contact Lens.

[11] Bonini S, Rama P, Olzi D (2003). Neurotrophic keratitis. Eye (Lond).

[12] Dhillon VK, Elalfy MS, Al-Aqaba M (2014). Anaesthetic corneas with intact sub-basal nerve plexus. Br J Ophthalmol.

[13] Ferrari G, Chauhan SK, Ueno H (2011). A novel mouse model for neurotrophic keratopathy: trigeminal nerve stereotactic electrolysis through the brain. Invest Ophthalmol Vis Sci.

[14] Nishida T, Yanai R (2009). Advances in treatment for neurotrophic keratopathy. Curr Opin Ophthalmol.

[15] Pradhan E, Khatri A, Ahmed AA, Lama AJ, Khanal R, Bajracharya L, Adhikari S (2020). Lightning injury to eye: brief review of the literature and case series. Clin Ophthalmol.

[16] Labetoulle M, Baudouin C, Calonge M (2019). Role of corneal nerves in ocular surface homeostasis and disease. Acta Ophthalmol.

[17] Aggarwal S, Kheirkhah A, Cavalcanti BM (2015). Autologous serum tears for treatment of photoallodynia in patients with corneal neurpathy: efficacy and evaluation with in vivo confucal microscopy. Ocul Surf.

[18] Mastropasqua L, Massaro-Giordano G, Nubile M (2017). Understanding the pathogenesis of neurotrophic keratitis: the role of corneal nerves. J Cell Physiol.

